# [Retracted] miR‑23a suppresses pancreatic cancer cell progression by inhibiting PLK‑1 expression

**DOI:** 10.3892/mmr.2025.13475

**Published:** 2025-02-25

**Authors:** Bin Chen, Akao Zhu, Lei Tian, Ying Xin, Xinchun Liu, Yunpeng Peng, Jingjing Zhang, Yi Miao, Jishu Wei

Mol Med Rep 18: 105–122, 2018; DOI: 10.3892/mmr.2018.8941

Following the publication of this paper, and subsequently to the publication of a corrigendum (DOI: 10.3892/mmr.2022.12738) that was intended to address the issue of a pair of duplicated data panels in Fig. 2C, it was drawn to the Editor's attention by a concerned reader that there regrettably remained issues with overlapping data panels in the revised version of Fig. 2 that was provided by the authors; furthermore, the revised data that were included in the corrected version of this figure were found to have appeared previously in a paper published in the journal *OncoTargets and Therapy* featuring different authors at different research institutes, which has subsequently been retracted.

In view of the fact that the abovementioned data had already apparently been published prior to its submission to *Molecular Medicine Reports*, the Editor has decided that this paper should now be retracted from the Journal. The authors were asked for an explanation to account for these concerns, but the Editorial Office did not receive a satisfactory reply. The Editor apologizes to the readership for any inconvenience caused.

